# Perovskite Solar Cells: A Review of the Latest Advances in Materials, Fabrication Techniques, and Stability Enhancement Strategies

**DOI:** 10.3390/mi15020192

**Published:** 2024-01-27

**Authors:** Rakesh A. Afre, Diego Pugliese

**Affiliations:** 1 Centre of Excellence in Nanotechnology (CoEN), Faculty of Engineering, Assam down town University (AdtU), Guwahati 781026, Assam, India; 79.rakesh@gmail.com; 2 National Institute of Metrological Research (INRiM), Strada delle Cacce 91, 10135 Torino, Italy

**Keywords:** perovskite solar cells, photovoltaic technology, power conversion efficiency, film fabrication, stability and scalability, lead-free alternatives, tandem devices, environmental impact

## Abstract

Perovskite solar cells (PSCs) are gaining popularity due to their high efficiency and low-cost fabrication. In recent decades, noticeable research efforts have been devoted to improving the stability of these cells under ambient conditions. Moreover, researchers are exploring new materials and fabrication techniques to enhance the performance of PSCs under various environmental conditions. The mechanical stability of flexible PSCs is another area of research that has gained significant attention. The latest research also focuses on developing tin-based PSCs that can overcome the challenges associated with lead-based perovskites. This review article provides a comprehensive overview of the latest advances in materials, fabrication techniques, and stability enhancement strategies for PSCs. It discusses the recent progress in perovskite crystal structure engineering, device construction, and fabrication procedures that has led to significant improvements in the photo conversion efficiency of these solar devices. The article also highlights the challenges associated with PSCs such as their poor stability under ambient conditions and discusses various strategies employed to enhance their stability. These strategies include the use of novel materials for charge transport layers and encapsulation techniques to protect PSCs from moisture and oxygen. Finally, this article provides a critical assessment of the current state of the art in PSC research and discusses future prospects for this technology. This review concludes that PSCs have great potential as a low-cost alternative to conventional silicon-based solar cells but require further research to improve their stability under ambient conditions in view of their definitive commercialization.

## 1. Introduction

Among the most plentiful and environmentally friendly renewable energy sources, solar energy has the ability to both lessen the environmental effects of fossil fuels and supply the world’s growing electricity needs. Still, there are a number of obstacles facing modern photovoltaic (PV) technologies, including high costs, poor efficiency, inconsistent performance, and environmental concerns. Consequently, in order to circumvent these restrictions and allow for the widespread use of solar cells, new materials and technologies must be created. Given their remarkable advancement in power conversion efficiency (PCE), which has increased from 3.5 to 25.8% in just ten years, perovskite solar cells (PSCs) have emerged as a promising candidate for the next generation of PV technology [1,2]. PSCs are made up of a layer of perovskite materials, which are hybrid organic–inorganic compounds with the general formula ABX_3_, where X is a halide anion (like iodide, bromide, or chloride), A is a monovalent cation (like methylammonium, formamidinium, or cesium), and B is a divalent metal (like lead or tin). The perovskite layer is positioned between two electrodes, usually a transparent conductive oxide (TCO) and a metal, and two charge transport layers (CTLs), typically an electron transport layer (ETL) and a hole transport layer (HTL). PSCs operate on a similar principle as conventional dye-sensitized solar cells (DSSCs) or organic solar cells (OSCs), in which light absorption by the perovskite layer produces electron–hole pairs (excitons), which are subsequently transported to the electrodes and separated by the electric field at the perovskite/CTL interfaces, producing an open-circuit voltage (*V_oc_*) and a photocurrent. Compared to DSSCs and OSCs, PSCs have a number of advantages, including a higher absorption coefficient, a longer diffusion length, a lower rate of recombination, and a higher degree of defect tolerance. These factors raise PCE because they increase both the *V_oc_* and the short-circuit current density (*J_sc_*) [3,4].

**Figure 1:**
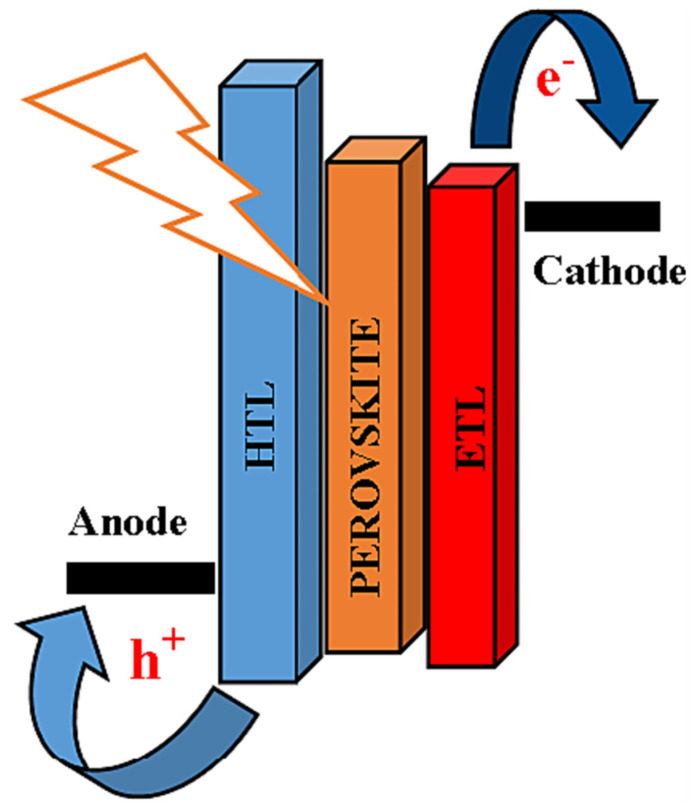
Working principle of PSCs. Reproduced with permission from Ref. [6], Copyright 2023 Elsevier.

As anticipated above, a conventional PSC device consists of five fundamental layers: the conducting substrate (typically indium-doped tin oxide (ITO) or fluorine-doped tin oxide (FTO)), the HTL, the perovskite light-absorber layer, the ETL, and the metal electrode (mainly copper (Au) or silver (Ag)) [5]. When the solar cell is illuminated, the ETL/HTL extracts photogenerated electrons/holes from the perovskite absorber layer and transports them to the cathode/anode, as shown schematically in Figure 1 [6].

Limiting the analyses to the role of HTL in maximizing the PCE of the solar cell, the latter should exhibit highest occupied molecular orbital (HOMO) and lowest unoccupied molecular orbital (LUMO) energy levels properly aligned with those of the other device components, namely the perovskite absorber layer and the back electrode. Taking the vacuum condition as a reference, the HOMO level of HTL should be higher in energy than the perovskite HOMO so as to facilitate charge transportation. Secondly, the LUMO of the HTM should be as high as possible with respect to the perovskite LUMO, since this energy misalignment would avoid electrons moving from the perovskite to the HTL, thus reducing the recombination exerting an electron-blocking effect. Moreover, the HOMO level of the HTL should lie just below the Fermi level of the back electrode in order to assure a fast charge collection [7].

**Figure 2:**
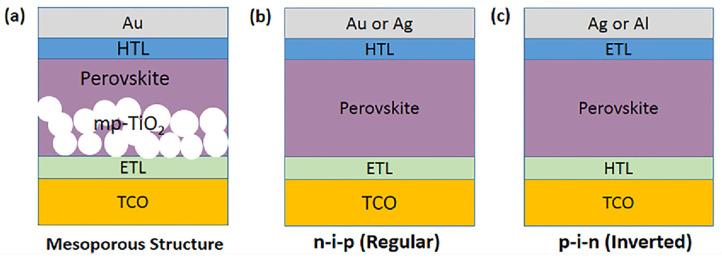
Device structures of PSCs: (**a**) mesoporous, (**b**) planar n-i-p (regular), and (**c**) planar p-i-n (inverted). Reproduced with permission from Ref. [10], Copyright 2016 American Chemical Society.

In recent decades, two typical PSC structures have been proposed, i.e., mesoporous and planar [8] (see Figure 2). The mesoporous structure consists of an FTO/ITO substrate, a hole blocking layer, a scaffold that can be either conductive TiO_2_ or insulating Al_2_O_3_, a perovskite absorber, an HTL, and the top metal contact electrode. Planar PSCs are less difficult to manufacture due to the absence of high-temperature processes and are commonly classified into two types based on the location of charge transporting layers in the devices: conventional n-i-p structures and inverted p-i-n structures [8]. In conventional planar n-i-p type perovskite devices, the HTL is located between the perovskite layer and the metal electrode (Ag or Au) and the ETL layer is deposited on the bottom transport layer. However, in inverted planar p-i-n structured PSCs the depositions of HTL and ETL are inverted. In inverted p-i-n PSCs, the electrons are extracted by electron transporting materials from the perovskite layer and transported into the metal electrode [9].

Even with PSCs’ outstanding accomplishments, there are still plenty of obstacles to overcome and chances to advance the development and application of this technology. Among the principal difficulties, it is worthwhile mentioning:

(i) Stability: PSCs’ performance and lifespan can be negatively impacted by exposure to moisture, oxygen, light, heat, and mechanical stress. The primary causes of PSC instability are the interfacial reactions between the perovskite and CTLs, the intrinsic instability of the perovskite materials, and the deterioration of the electrodes and encapsulation materials [11];
(ii) Scalability: PSCs are primarily made using solution-based techniques like inkjet printing and spin coating, which work well for small-area devices but not for large-area modules. Therefore, the development of scalable fabrication techniques that can produce high-quality, uniform perovskite films and devices over large areas is required. Examples of these techniques include roll-to-roll processing, doctor blading, slot–die coating, and spray coating;
(iii) Toxicity: Lead, which is a common ingredient in PSCs, is a heavy metal that is toxic and poses major health and environmental risks. Thus, it is necessary to reduce the amount of lead by alloying it with other metals or using mixed-halide perovskites, or to replace lead with less toxic or non-toxic alternatives like tin, bismuth, or antimony.

The following are some of the primary avenues for PSCs development:

(i) Tandem cells: By varying the perovskite materials’ composition, PSCs can exhibit a band gap that is tunable. In order to harvest a wider spectrum of solar radiation and achieve higher PCE, this allows for the fabrication of tandem or multi-junction cells, where two or more PSCs with different band gaps are stacked on top of each other, or on top of a silicon or thin-film solar cell;
(ii) Flexible and wearable devices: Using low-temperature and solution-based techniques, PSCs can be fabricated on flexible substrates like plastic or metal foils. This allows for the integration of PSCs with wearable electronics like smart watches, sensors, or displays, as well as the creation of flexible and lightweight solar modules for a variety of uses [12,13,14];
(iii) Perovskite light-emitting diodes (LEDs) and lasers: PSCs can also function as light-emitting devices by introducing electrons and holes into the perovskite layer through an applied voltage; within this layer, electrons and holes recombine and emit light. High brightness, color purity, and tunability have been demonstrated by perovskite LEDs and lasers, which can be used for illumination, displays, or communication.

A thorough analysis of the latest developments and future prospects of PSCs is presented in this review, addressing the following topics: (1) the structural and fundamental characteristics of perovskite materials and their influence on PSC performance; (2) the synthesis techniques and optimization strategies of perovskite films and devices; (3) the stability concerns and methods for enhancing PSCs; (4) the challenges and solutions related to PSC scalability; (5) the toxicity concerns and mitigation strategies related to PSCs; and (6) the newly emerging applications and innovations of PSCs. In addition to providing scholars and industry professionals in the PSC field with a helpful resource, our review should stimulate fresh thinking and new avenues for the advancement of this exciting technology.

## 2. Materials

### 2.1. Perovskite Absorber Materials

#### 2.1.1. Crystal Structure and Electronic Properties of Perovskites

Perovskites have the general chemical formula ABX_3_, where A and B are different sized cations and X is an anion (usually oxygen or a halide). The term “perovskite” comes from the calcium titanate (CaTiO_3_) mineral, which was the first compound with this structure to be discovered [15]. The remarkable physical properties of perovskites, such as their photovoltaic activity, magnetism, ferroelectricity, and superconductivity, have led to a notable surge in interest in these materials in recent times. In particular, organic A- and/or X-site hybrid organic–inorganic perovskites have grown in popularity as low-cost, high-efficiency solar cell alternatives [16].

The X anions in the face centres, the B cations in the centre, and the A cations in the unit cell corners occupy the cubic symmetry of the ideal perovskite structure. The A cations are in a 12-fold coordination with the X anions, which are surrounded by an octahedron of X anions around the B cations. The stability of the perovskite crystal structure is determined by two important empirical parameters, the octahedral factor (*µ*) and the Goldschmidt tolerance factor (*t*), which are defined as follows:

(1) $$\mu =\frac{r_{B}}{r_{X}}$$

(2) $$t=\frac{r_{A}+r_{X}}{\sqrt{2}\left(r_{B}+r_{X}\right)},$$

where ionic radii of the A, B, and X ions are denoted by *r*_A_, *r*_B_, and *r*_X_, respectively. The degree of distortion of the octahedron is measured by the octahedral factor, whereas the tolerance factor gauges the degree of fit between the A cation and the BX_6_ octahedron. In addition, *t* = 1 and *μ* = 1 represent the ideal perovskite structure, while deviating from these values signifies instability and distortion in the structure.

Perovskite structures are generally stable at 0.8 < *t* < 1.1 and 0.44 < *μ* < 0.94. While the cubic perovskite’s ideal *t* value is 1, most perovskites actually have lower symmetries, such as tetragonal, orthorhombic, rhombohedral, or mono-clinic, and deviate from this ideal structure. These distortions are frequently linked to the development of ferroelectric or anti ferroelectric phases, which, respectively, have a periodic alternation of electric polarization and spontaneous electric polarization [17].

A few examples of perovskites with different crystal structures are summarized in Table 1. The well-known ferroelectric perovskite barium titanate (BaTiO_3_) changes phases from cubic to tetragonal to orthorhombic to rhombohedral as temperature drops. Strontium titanate (SrTiO_3_) is a cubic perovskite at room temperature, but it becomes a superconductor at low temperatures. The hybrid perovskite CH_3_NH_3_PbI_3_ has a cubic structure at high temperatures and a tetragonal structure at room temperature [18]. It is one of the perovskites for solar cell applications that has been studied the most.

The electronic characteristics of perovskites are mainly determined by the B cations and X anions that make up the conduction band (CB) and valence band (VB). Although the A cations have little effect on the electronic structure, they can change the dielectric constant and lattice parameters to affect the band gap and carrier transport. Perovskites’ band gap can be adjusted by varying the composition and structure of the material. For example, in the rhombohedral phase of BaTiO_3_, the band gap drops from 3.2 eV in the cubic phase to 2.7 eV because of hybridization of the Ti 3d and O 2p orbitals and an increase in the Ti-O-Ti bond angle [15]. Decreasing the Pb-I-Pb bond angle and increasing the lattice constant cause CH_3_NH_3_PbI_3_’s band gap to go from 1.6 eV in the tetragonal phase to 1.5 eV in the cubic phase. Changing the B and/or X ions with other elements allows perovskites to have a customized band gap. In CH_3_NH_3_PbX_3_ (X = Cl, Br, I), for instance, the band gap increases from 1.5 up to 2.3 eV as the halide transitions from iodide to chloride. This is because the X ion’s electronegativity and ionic radius decrease. By changing the elements that the B and/or X ions are made of, perovskites can also be tuned [18].

Perovskites’ carrier transport is influenced by defects like vacancies, interstitials, or impurities that can serve as charge carrier traps or recombination sites. Stochastic deviations or thermal disorders of the material are the sources of intrinsic defects; contamination or doping of the material are the cause of extrinsic defects. The energy levels and defect concentration can be regulated by the synthesis conditions, which include temperature, pressure, solvent, and precursor ratio. In order to increase carrier collection and decrease recombination losses, defect engineering is a crucial tactic for PSC performance. Some examples of the electrical properties of perovskites are shown in Table 2. The band gap, defect density, and carrier mobility of a few representative perovskites are compared in the table along with their solar cell efficiencies. Higher carrier mobilities, lower defect densities, and smaller band gaps in the hybrid perovskites compared to the oxide perovskites translate into higher solar cell efficiencies. One issue that needs to be resolved, though, is the defect density in perovskite materials. The term “defect density” describes the existence of flaws or abnormalities in the perovskite materials’ crystal lattice structure. Perovskite solar cells’ stability and performance may suffer as a result of these flaws.

Elevated recombination rates of charge carriers can result from high defect densities in perovskite materials, thereby diminishing the solar cell’s overall efficiency. Perovskite-based solar cells and light emitters have shown exceptional efficiency despite the high defect density in perovskite materials [20]. One suggestion for mitigating the high defect density in PSCs is to employ organic HTLs. More specifically, they reduce contact with perovskite material defects by acting as a buffer layer between the electrode and the perovskite active layer. Device performance is improved by this buffer layer, which also suppresses recombination and aids in charge carrier extraction. The organic HTL in PSCs provides additional benefits besides reducing the effects of high defect density. Better stability, enhanced compatibility with other layers in the device, and improved charge transport properties are all provided by it.

Perovskites are a broad class of materials possessing a range of electrical characteristics and crystal forms. They have been thoroughly studied for many applications, most notably solar cells, where they have quickly attained remarkable efficiencies. The primary obstacles to further perovskite material development are comprehending the underlying mechanisms of physical phenomena, enhancing stability and reliability, and resolving safety and environmental concerns [21].

#### 2.1.2. Composition Engineering and Band Gap Tuning of Perovskites

Composition engineering is a tempting technique for enhancing perovskite’s structure because it can directly alter the material’s intrinsic qualities. As a result, perovskites of higher quality and greater photovoltaic performance can be produced [22,23,24]. A brief synopsis of the compositional changes in record perovskite devices over time is given in [22,23,24]. Additives can be classified into two categories: inorganic additives, which include metal cations and inorganic acids, and organic additives, which include polymers, small molecules, and fullerenes. Various additives possess the capability to control the perovskite crystallization process, leading to enhanced stability. Usually, organic molecules do not change the material’s intrinsic properties; instead, they only deactivate surface defect states. Conversely, the density of traps in the bulk could be lowered by adding inorganic molecules to the lattice. A, B, and/or X ion substitutions can be used to engineer the composition of perovskite, affecting the band gap, carrier transport, crystal structure, and material stability. The A cations can be organic (methylammonium [MA], formamidinium [FA], guanidinium [GA], and so on) or inorganic (Cs, Rb, K, Na, and so forth). Cl, Br, or I can be utilized as X anions, and Pb or Sn as B cations. The choice of A, B, and X ions can produce different types of perovskites, such as three-dimensional (3D), 2D, quasi-2D, or 0D, depending on the dimensionality of the BX_6_ octahedral network. A little composition engineering of perovskites is shown in Table 3. With a 1.73 eV band gap, CsPbI_3_ is a cubic 3D perovskite [25]. Nevertheless, it is thermally unstable and breaks down into CsI and PbI_2_ at room temperature. The perovskite’s band gap can be adjusted by partially substituting MA or FA for Cs. For instance, MAPbI_3_ has a tetragonal structure and a band gap of 1.55 eV, while FAPbI_3_ has a hexagonal structure [26]. Since Sn exhibits a larger ionic radius and less electronegativity than Pb, it is possible to further reduce the band gap of the perovskite by partially substituting Sn for Pb. For instance, MASnI_3_ has a band gap of 1.3 eV and an orthorhombic structure. Conversely, oxidation-prone Sn-based perovskites need cautious synthesis and processing conditions. Because Br and Cl have smaller ionic radii and higher electronegativities than I, partially substituting Br or Cl with I can increase the band gap of the perovskite [27]. For instance, the band gap of MAPbBr_3_ is 2.3 eV, whereas the band gap of MAPbCl_3_ is 3.1 eV, indicating a tetragonal structure. Mixed-halide perovskites, on the other hand, experience halide segregation—that is, the phase separation of the perovskite into regions with low and high band gaps—under light illumination [28].

For the development of efficient perovskite-based devices, such as solar cells, perovskite engineering and band gap tuning are crucial. These techniques involve changing the perovskite materials’ composition to enhance their performance, stability, and adaptability. The iodine and bromine ratios in the perovskite structure can be adjusted by researchers to fine-tune the material’s band gap, which determines its capacity to absorb and convert sunlight into electricity [32]. Moreover, halide ions and cations can be exchanged or replaced using perovskite engineering techniques, providing perovskite materials an even greater control over their band gap and other characteristics. By utilizing composition engineering and band gap tuning, researchers aim to get around the instability and toxicity of Pb-based perovskites and create stable, high-performing perovskite devices that can be used in photovoltaics, optoelectronics, and other fields [33,34]. Band gap tuning and perovskite engineering offer a promising path toward the creation of effective and adaptable perovskite-based devices. With the use of these methods, scientists can modify the band gap and other characteristics of perovskite materials to best suit their intended uses. By carefully adjusting the composition of perovskite materials, researchers can increase the efficiency of perovskite solar cells. Tin (Sn) or germanium (Ge), for example, can be used to replace lead (Pb) to lessen PSC toxicity and its negative effects on the environment [35]. It is possible to adjust the band gap and absorption spectrum of PSCs by mixing various halides, such as iodide, bromide, or chloride. The energy levels and wave functions of the VBs and CBs are impacted by changing the size and shape of the BX_6_ octahedra, which is how perovskite band gap tuning is achieved. By adding imperfections or impurities to the perovskite material, which result in localized states within the band gap, the band gap can also be adjusted [36].

The best performing solar cells to date have largely used perovskite materials with band gaps in the range of 1.48–1.62 eV [37,38]. On the other hand, a wider range of the solar spectrum must be harvested by materials with smaller band gaps. This is particularly crucial when creating effective tandem solar cells, which have the capacity to surpass the basic Shockley-Queisser limit on a single junction solar cell’s efficiency [39]. High-efficiency solar cells on flexible, lightweight substrates can be produced at low temperatures thanks to all-perovskite tandem solar cells. However, there is no way to lower the band gap below 1.48 eV using simple halide substitution. Lower band gaps have been achieved by cation substitution techniques, most notably by substituting tin for the lead cation on the B-site [40]. Based on this approach, a mono-lithic all-perovskite tandem solar cell with near-optimal band gaps that included a tin-lead perovskite absorber as the bottom cell was recently demonstrated. Moreover, even though partial halide substitution allows for a greater range of band gaps in the top cells of tandems, many of these blends are not photostable and show the Hoke effect, which is the segregation of the mixture into domains rich in iodide and bromide upon exposure to light [41,42]. This effect makes many high-gap materials unable to attain high *V_oc_* in solar cells. For wide-gap perovskite materials, therefore, alternative band gap tuning strategies are also crucial.

By adjusting the material’s composition and structure, perovskites’ band gap can be adjusted. Achieving color-tunable perovskite LEDs and maximizing the performance of PSCs depend heavily on band gap tuning. A variety of techniques can be employed to adjust the band gap, such as strain engineering, cation alloying, halide mixing, and dimensionality control. Depending on the degree of cation disorder, the process of combining various A or B cations in a perovskite structure is known as cation alloying. This can lead to a continuous or discrete change in the band gap. By combining Cs and MA or FA in the A-site, for example, the perovskite’s band gap can be continuously tuned from 1.73 eV (CsPbI_3_) to 1.55 eV (MAPbI_3_) or 1.48 eV (FAPbI_3_). By combining Pb and Sn in the B-site, the band gap of the perovskite can be discretely tuned, with intermediate values of 1.40 eV (MAPb_0.5_Sn_0.5_I_3_) and 1.35 eV (MAPb_0.25_Sn_0.75_I_3_). The band gap ranges from 1.55 eV (MAPbI_3_) to 1.30 eV (MASnI_3_). Depending on the halide ratio, the process of mixing various X anions in the perovskite structure—known as halide mixing—can cause a continuous change in the band gap. For instance, the band gap of the perovskite can be continuously tuned from 1.55 eV (MAPbI_3_) to 2.30 eV (MAPbBr_3_) by combining I and Br in the X-site. Intermediate values of 1.75 eV (MAPbI_0.5_Br_0.5_) and 1.90 eV (MAPbI_0.25_Br_0.75_) can also be achieved [43,44,45,46].

#### 2.1.3. Lead-Free and Low-Toxicity Perovskites

Since lead (Pb) is the primary component of the light-absorbing layer, there are significant worries about the effects PSCs will have on the environment and human health as well as the future availability and affordability of Pb [47]. Because of this, creating lead-free, low-toxicity perovskite materials is a crucial task for the long-term, widespread application of PSCs. With its excellent optoelectronic properties, including a high absorption coefficient, a long charge carrier diffusion length, a low trap density, and a tunable band gap, methylammonium lead trihalide (MAPbX_3_) is the most studied perovskite for photovoltaic solar cells [48]. In contrast, Pb is a highly toxic element that can damage the kidneys, reproductive organs, nervous system, and cause pollution and bioaccumulation in the environment. Additionally, Pb is a relatively expensive and scarce metal that is in high demand across a range of industries due to its limited supply [47]. In order to allay these worries, a number of methods for substituting different elements or compounds for lead in the perovskite structure have been put forth. The objective is to achieve lower toxicity and cost while maintaining performance and stability that is on par with or better than before. Four types of strategies can be employed to replace lead: (i) substituting other metal cations for lead; (ii) substituting metal clusters or molecular complexes for lead; (iii) substituting organic cations for lead; and (iv) substituting non-metal anions for lead [49].

**Figure 3:**
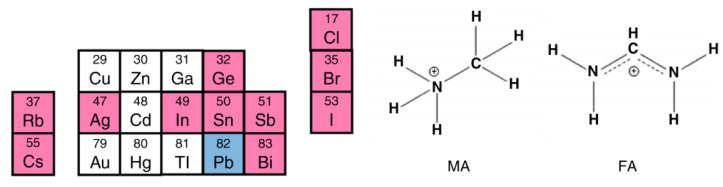
A portion of the periodic table that shows some of the possible elements that were taken into consideration for lead-free perovskites, such as metals and halides (Cl, Br, I), A-site cations (Rb, Cs), and the molecular structures of formamidinium (FA) and methylammonium (MA). Adapted from Ref. [50] under the Creative Common license CC BY.

The perovskite structure can be modified by substituting Pb with other metal cations, such as tin (Sn), bismuth (Bi), or germanium (Ge), which have comparable ionic radii and valence states (see Figure 3). Nevertheless, these substitutes possess certain drawbacks, including elevated toxicity (for Sn and Ge), reduced abundance (for Ge and Bi), and inadequate stability (for Sn and Ge) [50]. Because they can form a solid solution with Pb to tune the optoelectronic properties and improve stability, and because they have a band gap and absorption coefficient similar to Pb-based perovskites, Sn-based perovskites have attracted the most attention among them. On the other hand, Sn-based perovskites experience a significant reduction in charge carrier mobility and lifetime due to the severe oxidation of Sn^2+^ to Sn^4+^. Consequently, a number of approaches to stop or lessen Sn oxidation have been developed. These include the use of passivation agents, additives, protective layers, and low-temperature processing. Utilizing a mixed-cation and mixed-halide perovskite with a trace of Pb and an organic passivation layer, the highest efficiency for Sn-based PSCs to date has been reported at 12.6% [51].

The photocurrent and efficiency of Ge-based perovskites are lower than those of Sn-based perovskites due to their larger band gap and lower absorption coefficient. Moreover, the scarcity and high cost of Ge restrict its availability and scalability for PSCs. With a trace of Pb and a hole-transporting layer, a mixed-cation and mixed-halide perovskite was used to achieve the highest efficiency of 7.3% for Ge-based PSCs to date. Compared to Sn- and Ge-based perovskites, Bi-based perovskites are less toxic and more stable, but they are not appropriate for single-junction PSCs because of their large band gap and low absorption coefficient [52,53]. However, in tandem PSCs, Bi-based perovskites can be employed as top cells, collecting high-energy photons while letting low-energy photons reach the bottom cell. A mixed-cation and mixed-halide perovskite with a trace of Pb and a silicon bottom cell was used to achieve the highest efficiency for Bi-based PSCs to date, which is 9.2% [54,55].

To remove Pb from perovskite, the first fundamental stage is to find a less toxic replacement for Pb. Here, the attention will be focused on recent advances in Pb-free perovskite light absorbing materials based on various substitutional metal elements, which have not been well summarized to date, owing to the very short history of lead-free PSCs and the dynamic advances of this research field in recent years. We sincerely hope that by reviewing the development of lead-free perovskites for PV applications, we can provide readers with new insights for further exploring new types of low-cost, low-toxicity, high-performance PSCs. The use of lead in the perovskite material is primarily responsible for the toxicity and environmental impact of PSCs. As a result, there is growing interest in developing lead-free, low-toxicity perovskites that can maintain or even improve PSC performance. Alternative elements studied for replacing lead in PSCs include tin (Sn), germanium (Ge), copper (Cu), antimony (Sb), and bismuth (Bi). These elements have chemical properties similar to lead, but they are less toxic and more abundant in nature. Lead-free perovskites, on the other hand, face some challenges, such as lower efficiency, lower stability, or more complex synthesis methods [50,56].

### 2.2. Charge Transport Materials

PSCs are made up of two charge transport layers (CTLs) that extract and transport photogenerated charge carriers from the perovskite layer to the electrodes. The properties and interfaces of the CTLs, which should exhibit good electrical conductivity, suitable energy level alignment, high transparency, and good compatibility with the perovskite layer, have a large impact on the performance and stability of PSCs. In this review, the recent progress and challenges of various CTLs for PSCs, including hole transport materials (HTMs) and electron transport materials (ETMs), are summarized and some perspectives for future research directions are also offered.

#### 2.2.1. Hole Transport Materials (HTMs)

Achieving long-term stability, however, is one of the most challenging tasks for PSCs because the perovskite layer is prone to deterioration by heat, light, oxygen, and moisture. Therefore, enhancing the functionality and endurance of PSCs depends heavily on the choice and construction of CTLs. As essential CTL, the hole transport layer (HTL) balances the energy levels of the anode and the perovskite layer, inhibits electron diffusion, and stops electron and hole recombination [57]. Metal oxides, carbon-based materials, and organic semiconductors have all been used to make HTLs for PSCs. It is worthwhile highlighting that organic HTMs display several advantages over their inorganic counterparts, including tunable molecular structures, adjustable energy levels, good compatibility with the perovskite layer, and low-temperature solution processing [58].

##### Organic HTMs

**Figure 4:**
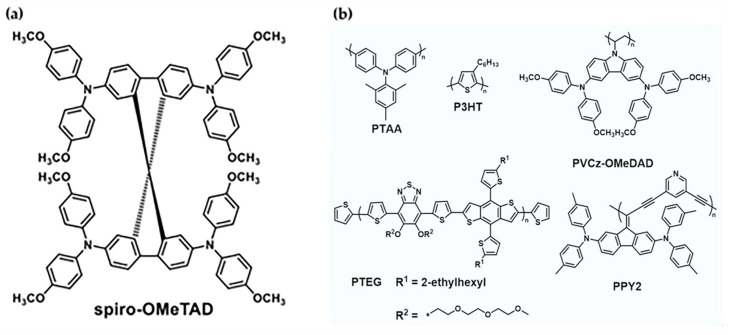
Examples of (**a**) small molecular organic and (**b**) polymer HTMs employed in PSCs. Adapted from Ref. [64] under the Creative Common license CC BY.

Organic HTMs are classified into two types: small molecular organic HTMs and polymer HTMs. Small molecular organic HTMs have well-defined structures that make purification and characterization easier. Furthermore, small molecular organic HTMs can form compact and uniform films on the perovskite layer, improving charge transport and extraction efficiency. Small molecular organic HTMs include Spiro-OMeTAD, Poly[bis(4-phenyl)(2,4,6-trimethylphenyl)amine] (PTAA), and their derivatives [59,60]. Polymer HTM structures are larger and more complex, allowing for greater functionalization and modification. Polymer HTMs can also form interpenetrating networks with the perovskite layer, increasing mechanical stability and decreasing interface defects. Polymer HTMs include Poly(3-hexylthiophene) (P3HT), Poly(3,4-ethylenedioxythiophene) poly(styrene sulfonate) (PEDOT:PSS), and their derivatives (see Figure 4). The molecular structure of organic HTMs determines their electronic and optical properties, as well as their interactions with the perovskite layer and the anode [61,62]. Organic HTMs should, in general, have a planar and rigid structure that allows for stacking and orbital overlap, which improves hole transport and extraction efficiency. Furthermore, organic HTMs should have a high ionization potential and a high-lying HOMO level, which can match the perovskite layer’s VB and the anode’s work function, reducing energy loss and recombination rate. Finally, to avoid parasitic absorption and interference with perovskite layer light harvesting, organic HTMs should exhibit low optical absorption in the visible range [63].

The stability, film formation, and solubility of organic HTM substituents can all be impacted. Generally speaking, processing HTL solutions at low temperatures should be possible because organic HTMs should dissolve in standard organic solvents. Additionally, the right substituents should be present in organic HTMs to encourage the formation of uniform, smooth films on the perovskite layer, which will enhance charge transport efficiency and contact quality. In order to prolong the life of PSCs, organic HTMs should also have stable substituents that are resistant to deterioration by heat, light, moisture, and oxygen [65,66]. Even with the tremendous advancements in organic HTMs for PSCs, certain obstacles and restrictions still exist. Organic HTM synthesis and molecular design are still labor-intensive, trial-and-error processes that involve a significant commitment of time and money. Therefore, further theoretical and computational methodologies are required to inform the logical design and optimization of organic HTMs. Organic HTMs’ stability continues to lag behind that of inorganic HTMs, especially in harsh environmental conditions. As a result, more effective approaches, such as the addition of additives or dopants, the inclusion of hydrophobic or cross-linking groups, or the application of protective coatings, are required to improve the stability of organic HTMs. There is still a lack of comprehension and control over the compatibility and synergy of organic HTMs with other PSC elements [64,67]. Consequently, in view of better understanding the bulk and interfacial characteristics of organic HTMs and optimizing PSC fabrication and device engineering, more thorough and organized research is needed. In summary, the advantages of organic hybrid materials over their inorganic counterparts, including their tunable molecular structures, adjustable energy levels, good compatibility with the perovskite layer, and low-temperature solution processing, make them promising candidates for HTLs in PSCs. Here, common design strategies and challenges of organic HTMs for PSCs are reviewed and recent advances in organic HTMs for PSCs are summarized, including small molecular organic HTMs and polymer HTMs. We anticipate that this review will offer some helpful viewpoints and insights for the synthesis and use of organic HTMs for PSCs [59,68].

##### Inorganic HTMs

It’s interesting to note that a number of inorganic HTLs exhibit desirable and adjustable energy alignment with the perovskite layer in addition to high transparency. They are thought to be desirable barrier materials that shield the perovskite layer. As a result, a lot of research has been performed on inorganic materials, especially oxides, for hole collection in PSCs [69]. However, because the perovskite layer is sensitive to heat, light, oxygen, and moisture, achieving long-term stability is one of the most challenging problems for PSCs [70]. Therefore, choosing and creating the charge transport layers—which are in charge of removing and moving photogenerated carriers from the perovskite layer to the electrodes—is essential to enhance PSCs functionality and longevity.

Inorganic HTMs exist in two different varieties: metal-based and non-metal-based. The most popular inorganic HTMs for PSCs are metal-based like NiO_x_, CuSCN, CuI, CuO_x_, CuAlO_2_, CuCrO_2_, CuGaO_2_, CuS, CuInS_2_, CuZnSnS_4_, and CuBaSnS_4_ due to their appropriate energy levels, high conductivity, and effective hole extraction ability [71]. Conversely, metal-based HTMs have a few disadvantages, including low solubility, high processing temperature, and high optical absorption. As alternative inorganic HTMs for PSCs, non-metal-based HTMs like MoO_x_, WO_x_, V_2_O_x_, and CrO_x_ have been studied due to their higher solubility, lower optical absorption, and lower processing temperature. However, also non-metal-based HTMs display a few drawbacks, including poor compatibility with the perovskite layer, high trap density, and low conductivity [72,73,74].

The synthesis and preparation processes of inorganic HTMs influence their electronic and optical properties, as well as their interactions with the anode and perovskite layer. These factors also affect the crystallinity, purity, and stoichiometry of these materials. Generally speaking, a number of techniques, such as sol-gel, hydrothermal, spin coating, spray coating, sputtering, evaporation, chemical vapor deposition, and atomic layer deposition, can be used to synthesize inorganic HTMs. The intended characteristics and the inorganic HTMs compatibility with the perovskite layer and anode dictate the synthesis and preparation techniques [75,76]. The energy levels of inorganic HTMs control how well they extract and move holes from the perovskite layer to the anode, as well as how likely they are to cause recombination and energy loss. As a general rule, inorganic HTMs should have a high ionization potential and a high-lying valence band maximum (VBM) level that minimizes energy loss and recombination rate by matching the VB of the perovskite layer and the anode’s work function. In order to prevent parasitic absorption and interference with the light harvesting of the perovskite layer, inorganic HTMs should also have a large band gap [77].

The hole mobility of inorganic HTMs controls their capacity to move holes from the perovskite layer to the anode and affects the stability and performance of the device. High hole mobility is generally desired in inorganic HTMs because it can enhance charge transport and extraction efficiency, raising the fill factor (FF) and short-circuit current of PSCs. In addition, stable hole mobility is necessary for inorganic HTMs to withstand deterioration from heat, light, moisture, and oxygen, extending the PSCs’ lifespan [78]. The inorganic HTMs’ film morphology affects the stability and performance of the device, as well as the quality of their interface and contact with the anode and perovskite layer. On the perovskite layer, inorganic HTMs should, in general, form a smooth, uniform film that can enhance charge transport efficiency and contact quality. Moreover, a compact and dense layer of inorganic HTMs should form on the perovskite layer. This film can prevent electron diffusion and thereby increase the stability of PSCs [79].

Notwithstanding notable advancements in inorganic HTMs for PSCs, certain obstacles and restrictions still exist. The complexity and cost of the synthesis and preparation processes limit the large-scale manufacturing and application of inorganic HTMs. As a result, simpler and less expensive techniques for the synthesis and preparation of inorganic HTMs for PSCs are required. Because the energy levels and hole mobility of inorganic HTMs are still not properly matched and tuned with the perovskite layer and anode, there is a large energy loss and low charge extraction efficiency. As a result, more efficient ways for modifying and improving the hole mobility and energy levels of inorganic HTMs for PSCs are required [80,81]. Therefore, to better understand and optimize the stability and film morphology of inorganic HTMs for PSCs, a more thorough and methodical investigation is needed. Last but not least, due to their advantages over their organic counterparts—such as high stability, high conductivity, and low cost—inorganic HTMs are promising candidates for HTLs in PSCs [82].

##### Carbon-Based HTMs

Carbon black, graphene, and carbon nanotubes (CNTs) are the three categories of carbon-based HTMs. Amorphous carbon in the form of carbon black is composed of nanometer-sized graphite particles. Because of its high conductivity, large surface area, and good compatibility with the perovskite layer, carbon black has been widely used as a conductive additive in batteries and electrodes. It can also be an effective HTM for PSCs. Conversely, carbon black has a few disadvantages, such as poor film formation, high optical absorption, and low hole mobility [83,84].

A two-dimensional sheet of sp^2^-hybridized carbon atoms arranged in a honeycomb lattice is known as graphene. Because of its exceptional qualities, which include high hole mobility, transparency, flexibility, and stability, graphene is a perfect HTM for PSCs. Graphene’s high sheet resistance, low solubility, and high cost are some of its disadvantages. Cylindrical graphene nanostructures with variable diameters and chirality are called CNTs. Because of their superior qualities—high hole mobility, high transparency, high flexibility, and high stability—CNTs are a desirable HTM for PSCs [85]. But CNTs have drawbacks as well, like high cost, low solubility, and high sheet resistance.

The synthesis and modification processes used to create carbon-based HTMs have an impact on their electrical and optical characteristics, as well as how they interact with the anode and perovskite layer. These factors ultimately determine the purity, structure, and functionality of these materials. Many synthesis methods, such as arc discharge, chemical vapor deposition, and hydrothermal process, can be used to create carbon-based HTMs. Moreover, a range of techniques, such as doping, functionalization, and hybridization, can be applied to carbon-based HTMs to enhance their solubility, conductivity, and suitability for the anode and perovskite layer [86,87]. The energy levels of carbon-based hybrid thermocouples affect their propensity to cause recombination and energy loss, as well as their capacity to extract and transfer holes from the perovskite layer to the anode. In order to reduce energy loss and recombination rate, carbon-based HTMs should, in general, have a high ionization potential and a high-lying Fermi level that can match the VB of the perovskite layer and the work function of the anode. Additionally, low optical absorption in the visible spectrum is a requirement for carbon-based HTMs in order to prevent parasitic absorption and interference with the light harvesting of perovskite layers [88].

Hole mobility is what determines the capacity of carbon-based HTMs to move holes from the perovskite layer to the anode and how they affect stability and performance of the device. High hole mobility is generally desired in carbon-based HTMs because it can enhance charge transport and extraction efficiency, raising the FF and short-circuit current of PSCs. To further prolong the life of PSCs, carbon-based HTMs ought to have stable hole mobility that is resistant to deterioration by heat, light, moisture, and oxygen. The morphology of carbon-based HTM films affects device stability and performance, as well as contact quality and interface properties with the anode and perovskite layer. Generally speaking, carbon-based HTMs should create a uniform, smooth film on the perovskite layer, enhancing charge transport efficiency and contact quality. Furthermore, the perovskite layer should form a compact and dense film of carbon-based HTMs, which can enhance PSC stability by preventing electron diffusion [89].

Despite significant advancements in carbon-based HTMs for PSCs, some challenges and limitations remain. Carbon-based HTM synthesis and modification processes remain difficult and costly, restricting large-scale production and application. As a result, simpler and less expensive approaches for the synthesis and modification of carbon-based HTMs for PSCs are required. Carbon-based HTMs’ energy levels and hole mobility are yet poorly matched and adjusted with the perovskite layer and anode, resulting in high energy loss and low charge extraction efficiency. As a result, enhanced strategies for tweaking and enhancing the energy levels and hole mobility of carbon-based HTMs for PSCs are necessary [90]. There is still a lack of control and improvement in the film morphology and stability of carbon-based HTMs, resulting in poor contact quality and a high rate of deterioration. Because of this, more thorough and methodical research is needed to examine and optimize the stability and film morphology of carbon-based HTMs for PSCs. Lastly, because they have advantages over their counterparts like high conductivity, good stability, and low cost, carbon-based HTMs are promising candidates for HTLs in PSCs [91].

#### 2.2.2. Electron Transport Materials (ETMs)

**Figure 5:**
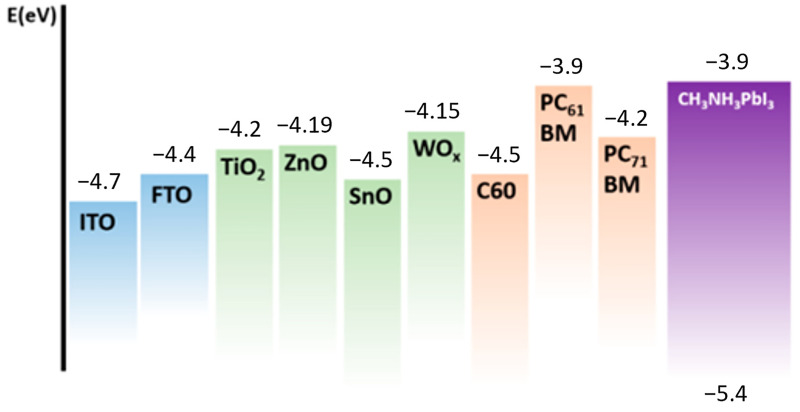
Energy levels of ETL materials and perovskite. Reproduced from Ref. [92] under the Creative Common license CC BY.

The electron transport layer (ETL), one of the CTLs, is essential for keeping the cathode and the perovskite layer’s energy levels in balance, inhibiting the diffusion of moisture and oxygen, and preventing electron-hole recombination. Metal oxides, CNTs, graphene, and organic semiconductors are just a few examples of the materials that have been successfully used as ETLs for PSCs. These materials present noticeable benefits, which include low-temperature solution processing, tunable molecular structures, and adjustable energy levels as shown in Figure 5.

##### Organic ETMs

Two categories of organic ETMs are commonly exploited in the field of PSCs: fullerene-based and non-fullerene-based. The most popular organic ETMs for PSCs are fullerene derivatives, such as C_60_, C_70_, and [6,6]-phenyl-C61-butyric acid methyl ester (PCBM), because of their excellent electron extraction ability, appropriate energy levels, and high electron mobility [93,94]. Fullerene-based ETMs do have certain disadvantages, though, like low stability, restricted solubility, and expensive cost. Non-fullerene organic ETMs have therefore grown in favor recently since they offer more variety and adaptability in terms of molecular design, synthesis, and modification. Non-fullerene organic ETMs come in two varieties: small molecular organic ETMs and polymer ETMs. The structures of small molecular organic ETMs are comparatively straightforward and well-defined, which facilitates their purification and characterization. Additionally, on the perovskite layer, small molecular organic ETMs can form uniform, compact films that enhance extraction efficiency and charge transport. Examples of small molecular organic ETMs are pyrazine-azaacene, naphthalene-diimide, perylene-diimide, and their derivatives. The larger and more complex structures typical of polymer ETMs enable greater functionalization and modification. Interpenetrating networks between polymer ETMs and the perovskite layer can enhance mechanical stability and minimize interface defects. Poly[(9,9-bis(3′-(N,N-dimethylamino)propyl)-2,7-fluorene)-alt-2,7-(9,9-dioctylfluorene)] (PFN) and Poly[(9,9-bis(3′-(N,N-dimethylamino)-co-2,7-(9,9-dioctylfluorene)] (PFNF) are examples of polymer ETMs [95].

The molecular structure impacts the electronic and optical characteristics of organic ETMs as well as how they interact with the cathode and perovskite layer. All things considered, the planar and stiff structure of organic ETMs should enable orbital overlap and stacking, which enhances the efficiency of electron transport and extraction. In addition, it is desirable for organic ETMs to possess a low-lying LUMO level and a high electron affinity. This combination can reduce energy loss and recombination rate by matching the cathode’s work function with the CB of the perovskite layer. In addition, organic ETMs ought to exhibit low visible optical absorption to prevent parasitic absorption and interference with the light harvesting of the perovskite layer [96].

The solubility, stability, and film formation of organic ETM substituents can be affected. Organic ETMs should generally dissolve in standard organic solvents, enabling the processing of ETL solutions at low temperatures. Moreover, suitable substituents for the formation of uniform and smooth films on the perovskite layer are important for improving charge transport efficiency and contact quality in organic electrochemical membranes. Additionally, stable substituents for organic ETMs that are resistant to deterioration from heat, light, moisture, and oxygen are needed to prolong the life of PSCs [97]. Even with the tremendous advancements in organic ETMs for PSCs, certain obstacles and constraints remain. To begin with, organic ETMs synthesis and molecular design are still labor-intensive, empirical processes that take a significant amount of time and money. As a result, more theoretical and computational techniques are required to inform the logical design and optimization of organic ETMs. Organic ETMs are less stable than inorganic ETMs, especially in harsh environmental conditions. As a result, more potent methods for increasing the stability of organic ETMs are required. Among these methods, it is worthwhile mentioning the incorporation of dopants or additives, the application of protective coatings, and the incorporation of hydrophobic or cross-linking groups. A little is currently known about how well organic ETMs integrate and interact with other PSC constituents. Consequently, in order to better understand the bulk and interfacial characteristics of organic ETMs and to develop and fabricate PSCs, more thorough and organized research is needed [92].

##### Inorganic ETMs

Inorganic ETMs are classified into two categories: metal oxides and non-metal oxides. Metal oxides like ZnO, SnO_2_, and TiO_2_ are the most often used inorganic ETMs for PSCs due to their good electron extraction ability, appropriate energy levels, and high electron mobility. Conversely, metal oxides have certain drawbacks, including high surface trap density, low conductivity, and processing at high temperatures. Because of this, non-metal oxides, including Cs_2_CO_3_, WO_3_, and MoO_3_, have been studied as potential substitute inorganic ETMs for PSCs because of their higher conductivity, lower processing temperature, and lower trap density. The two types of metal oxides and non-metal oxides can be further distinguished between compact and mesoporous. On the perovskite layer, compact ETMs deposit a thin, dense film that promotes good contact and low recombination rates. Mesoporous ETMs have a large surface area and high loading capacity because they form a thick, porous film on the perovskite layer. The perovskite layer’s deposition technique, along with the intended device architecture and performance, dictate the kind of ETMs that is utilized [98].

The procedures used for the synthesis and preparation of inorganic ETMs have an impact on their stoichiometry, crystallinity, and purity, all of which are related to their electrical and optical characteristics and interactions with the cathode and perovskite layer. All things considered, a number of methods, including sol-gel, hydrothermal, spin coating, spray coating, sputtering, evaporation, chemical vapor deposition, and atomic layer deposition, can be used to synthesize inorganic ETMs. The required qualities and the inorganic ETMs’ compatibility with the cathode and perovskite layer dictate the synthesis and preparation techniques that are employed.

The propensity of inorganic ETMs to cause recombination and energy loss, as well as their capacity to extract and transfer electrons from the perovskite layer to the cathode, are all determined by their energy levels. To reduce energy loss and recombination rate, inorganic ETMs should, in general, have a low-lying conduction band minimum (CBM) level and a high electron affinity that can match the CB of the perovskite layer and the cathode’s work function. In addition, a broad band gap is necessary for inorganic ETMs to prevent parasitic absorption and interference with the perovskite layer’s ability to harvest light [99]. Electron mobility of inorganic ETMs controls their capacity to move electrons from the perovskite layer to the cathode and affects device stability and performance. High electron mobility is generally desired in inorganic ETMs because it can enhance charge transport and extraction efficiency, raising the FF and short-circuit current of PSCs. To further prolong the life of PSCs, inorganic ETMs should possess stable electron mobility, which will shield them from deterioration by heat, light, moisture, and oxygen [100].

The quality of contact and interface properties with the perovskite layer and cathode, along with the stability and performance of the device, are influenced by the morphology of the inorganic ETM film. To enhance charge transport efficiency and contact quality, inorganic ETMs should, in general, deposit a uniform, smooth film on the perovskite layer. In addition, the formation of a compact and dense film by inorganic ETMs on the perovskite layer is expected to enhance PSC stability by preventing the diffusion of moisture and oxygen. Although inorganic ETMs for PSCs have advanced significantly, there are still certain obstacles and restrictions. The complexity and cost of inorganic ETMs’ synthesis and preparation processes limit their large-scale production and application [101]. As a result, less complex and costly techniques for the synthesis and preparation of inorganic ETMs for PSCs are required. The inorganic ETMs’ energy levels and electron mobility are not properly matched and optimized with the perovskite layer and cathode, resulting in high energy loss and poor charge extraction efficiency. More efficient methods of adjusting and enhancing the energy levels and electron mobility of inorganic ETMs are thus required for PSCs. The morphology and stability of inorganic ETM films are still poorly controlled and improved, resulting in poor contact quality and a high rate of degradation. As a result, in order to better understand and enhance the stability and film morphology of inorganic ETMs for PSCs, more thorough and methodical research is needed [102].

### 2.3. Electrode Materials

#### 2.3.1. Transparent Electrodes

Transparent conductive oxides (TCOs), such as ITO and FTO, are the most widely used bottom electrode in PSCs owing to their balanced conductivity and transparency [103]. ITO is more versatile, allowing for better transparency and resistance control and can be deposited by a wide variety of deposition methods on different substrates, while FTO requires high deposition temperature and toxic gas handling during its production process [104]. More importantly, ITO exhibits a smoother surface and higher transparency at a given conductivity compared with FTO. However, a significant disadvantage of ITO is its poor compatibility with high-temperature heat treatments, which significantly reduce its electrical conductivity [105]. Therefore, ITO has been employed by most of the inverted p-i-n structured or n-i-p planar structured PSCs in combination with low-temperature processed transport layers. It is worth mentioning that ITO has also been used as both the top and back electrode in planar n-i-p semitransparent PSCs employing SnO_2_, FAPbBr_3_ and PTAA as the ETL, perovskite absorber layer and HTL, respectively [106]. On the other hand, FTO has become the substrate of choice for mesoporous PSCs [104].

Despite the promising optical and electrical properties of TCO electrodes, their brittleness and high manufacturing costs have recently prompted researchers to develop various potentially low-cost alternative transparent conductors as the bottom electrode for PSCs [107,108,109,110].

Liu et al. [107] reported a new structure of PSC employing a Cd_2_SnO_4_ TCO substrate as the front electrode. Thanks to its higher electrical conductivity, optical transmission in the visible range, and lower surface roughness as compared to those of FTO, a higher PCE of 15.58% for the Cd_2_SnO_4_-based PSC was achieved.

Jin et al. [108] developed a novel silver nanowire (AgNW) composite electrode with an antioxidant-acid-modified chitosan polymer using a low-temperature solution process. The as-prepared electrode showed a root mean square (RMS) surface roughness lower than 10 nm over a large scan size of 50 μm × 50 μm and a remarkable long-term stability over an aging of 40 days at 85 °C in air. As a result, the PSC employing the composite as the bottom electrode exhibited a PCE of 7.9%, which corresponds to nearly 75% of that of the reference device with an ITO electrode.

Zhou et al. [109] fabricated a 1D-2D AgNWs-graphene (AgNWs-G) transparent electrode to be used in PSCs in place of the conventional ITO electrode. The optimized AgNWs-G film exhibited a relatively high transmittance of ~86% and a noticeable stability during the entire duration of air exposure, even at high temperatures or continuous illumination. Interestingly, the PSC assembled with this electrode showed an outstanding PCE of 15.31% and a long-term stability of 87.5% for 60 days in air conditions.

Zhu et al. [110] reported the fabrication of a biodegradable and biocompatible transparent conductive electrode derived from bamboo for flexible PSCs. The conductive bioelectrode highlighted extremely flexible and light-weight properties and allowed obtaining a record PCE of 11.68%, the highest among all reported biomass-based PSCs. Even more interestingly, this flexible device displayed a highly bendable mechanical stability, maintaining over 70% of its original PCE during 1000 bending cycles at a 4 mm curvature radius.

#### 2.3.2. Metal Electrodes

Metals are often used as the back electrode materials in PSCs due to their high conductivity and light reflectivity. Although excellent device efficiencies have been achieved using gold (Au) and silver (Ag) as back electrodes, their high cost and the significant device degradation arising from the reactions between the perovskite absorber and these metals have pushed researchers to explore alternative materials [111].

Among them, it is worthwhile mentioning less expensive metals such as copper (Cu), aluminum (Al), nickel (Ni), molybdenum (Mo), tungsten (W), etc., some non-metallic electrodes such as carbon and TCOs, and also some conductive polymers [111].

##### Gold (Au)

Gold, as a noble metal, is by far one of the most common and effective electrode materials for high-performance perovskite devices. Its work function matches well with the typically employed HTLs, such as CuSCN or Spiro-OMeTAD, or NiO_x_. The maximum PSC efficiency of 25.2% was achieved using a 100 nm-thick Au electrode deposited by thermal evaporation. Yoo et al. [112] comparatively investigated the most commonly used metal electrodes in PSCs to date and found that Au electrode, although being expensive [113], can be considered as the best choice to provide the optimal efficiency and stability for cell implementation.

Fan et al. [114] fabricated a transparent ultrathin gold electrode with enhanced electrical and optical properties like conductivity and optical reflectance-scattering and used it on the top PSC with CH_3_NH_3_PbI_3−x_Cl_x_ as the light collector in a tandem structured device. The four-terminal tandem solar cell showed an efficiency of 14.8%, with PCE of 8.98% as the top-cell contribution and 5.82% as the bottom cell.

Yang et al. [115] reported a PSC with a PCE as high as 19.0% using a nano-porous Au film electrode in substitution of the most common thermally evaporated Au electrode. The use of the nano-porous Au electrode provided a simple, effective, and practical alternative to complex metal electrode deposition methods for fabricating high-performance PSCs, also helping to accelerate their industrial application.

##### Silver (Ag)

Ag is becoming increasingly popular as a back electrode material for PSCs as it is cheaper compared to Au. Unlike Au, it is commonly used as the cathode for efficient p-i-n structured PSCs because its work function better matches the LUMO energy level of the most typically used ETLs, thus forming an Ohmic contact.

In 2012, Lee et al. [116] used Ag as a PSC electrode for the first time, achieving a PCE of 10.9%. Here, thermally evaporated Ag was used as the anode, and the perovskite solar device was assembled with the architecture glass/FTO/TiO_2_/Al_2_O_3_/MAPb(I, Cl)_3_/Spiro-OMeTAD/Ag. Improvement in device performance has been reported by the thermal evaporation of Ag nano-powder (~20 nm) due to improved charge transport across the transport layer and electrode [117].

Trinh and Kim [118] reported a fully solution-processed fabrication of PSCs using Ag nanoparticle film as the back electrode by lamination method. Devices were fabricated with the architecture FTO/compact TiO_2_/mesoporous TiO_2_/CH_3_NH_3_PbI_3_/Spiro-OMeTAD/PEDOT:PSS/D-sorbitol/Ag nanoparticle film. To deposit the Ag nanoparticle film, the nanoparticle Ag inks were spin coated on a flexible polyethylene terephthalate (PET) substrate and subsequently annealed. During this process, the heating temperature was varied to optimize the sheet resistance and surface roughness of annealed Ag nanoparticle film. PSCs were fabricated under a relative humidity of 45% and exhibited a stabilized PCE of 10.03%.

##### Other Metals and Alloys

In recent decades, low-cost metals such as aluminum (Al) [119], copper (Cu) [120], nickel (Ni) [121], molybdenum (Mo) [122] and tungsten (W) [123] have been investigated as rear electrode materials for PSCs to replace Au and Ag.

In particular, Al, characterized by high electrical conductivity and low cost, is a suitable candidate for the back electrode material, especially in the case of PSCs with an inverted structure, due to its low work function. You et al. [119] first used Al electrode in a PSC with a device structure constituted of substrate/ITO/PEDOT:PSS/CH_3_NH_3_PbI_3−x_Cl_x_/PCBM/Al and obtained a PCE of 11.5% and 9.2% for devices fabricated on rigid and flexible substrates, respectively. More recently, the same group reported a moisture-induced perovskite grain growth technique to markedly improve the film quality, carrier mobility, and lifetime [124]. The fabricated devices showed a noticeable PCE of 17.1% with an FF of 0.80.

To improve the device stability, Wang et al. [125] introduced a thin polystyrene (PS) tunneling layer between the perovskite and electron transport layers, acting as an encapsulation layer with the aim to prevent carrier recombination at the cathode. As a result, this polymeric interlayer prevented perovskite film from being damaged by moisture. The Al-based device showed a *V_oc_* of 1.10 V, a *J_sc_* of 22.9 mA cm^−2^, an FF of 80.6%, and a PCE of 20.3%. Furthermore, Al-based devices without encapsulation mostly retained their initial performance after 120 h of exposure to 40% relative humidity (RH).

Recently, Chen et al. [126] reported a record stable efficiency value of 23.2% for blade-coated p-i-n structured PSCs using a low-cost benzylhydrazine hydrochloride (BHC) reducing agent. The optimized devices highlighted an outstanding operational stability with almost zero degradation after 1000 h of operation at maximum power point (MPP). Additionally, it is worthwhile mentioning that Cu inks and Cu pastes are commercially available, thus enabling the scalable fabrication of PSCs with Cu top electrodes [127,128].

Cu is widely used as an electrode material due to its relatively low cost, good electrical conductivity, and good oxidation resistance. Zhao et al. [127] used Cu as the back electrode in inverted-structure PSCs for the first time and highlighted its outstanding stability. They discovered the stability of Cu even in direct contact with MAPbI_3_ and the absence of Cu diffusion in perovskite under mild thermal aging conditions. More recently, the same group reported ITO/PTAA/perovskite/indene-C60 bisadduct/C_60_/bathocuproine (BCP)/Cu devices, where the C_60_/BCP layer could suffer less damage during the thermal evaporation of Cu [120]. Even more importantly, devices based on MAPbI_3_ and FA_0.4_MA_0.6_PbI_3_ with unsealed Cu electrodes could preserve their performance with low PCE loss for 20–30 days under 20–60% RH.

Ni, thanks to its well-matched work function and low cost, is another promising electrode material for PSCs. Jiang et al. [129] first reported a sputtering-coated Ni film onto the spiro-OMeTAD layer in a regular-structure mesoporous PSC. The devices employing Ni electrodes achieved a comparable PCE (10.4%) with respect to the cells with Au top electrode (11.4%).

Later, Ku et al. [121] used a mesoporous Ni film as a printable counter electrode for PSC, which showed a PCE of 13.6%. Meanwhile, this low-cost Ni-based PSC device could be reused by washing and reloading the perovskite material and the reused one still exhibited an acceptable PCE of 12.1%.

Bryant et al. [130] used a semi-transparent adhesive laminate Ni-based electrode in PSCs and achieved a PCE up to 15.5%, with a reference device using Au electrode showing a PCE of 16.7%. Hence, it can be envisioned that Ni works very well as an alternative to Au electrode in PSCs.

Mo, featured by a high work function and acceptable electrical conductivity, revealed to be an interesting low-cost electrode material for high-efficiency PSCs [123]. Additionally, Mo exhibits a higher chemical stability against reactions with halides and oxidation in air than Ag and Al, respectively [122].

Motivated by the advantages of Mo, Jeong et al. [122] first developed sputtered Mo as the back electrode for PSCs. By improving the quality of the perovskite films, they obtained an optimized PCE of 15.06%, comparable to the solar cells with electrodes made of Au. In addition, the Mo electrode also displayed superior mechanical properties and wear resistance compared to the Au electrode.

In another study, low-cost nonprecious metal (Cu, Ni, W, and Mo) films, fabricated by magnetron sputtering, were used to replace Ag film as the back electrode of PSCs. No electrode corrosion was observed in PSCs with the Ni, W, and Mo electrodes as compared to devices with Ag and Cu electrodes [123].

Some metal alloys are also promising counter electrode materials for high-performance PSCs [131,132]. Jiang et al. [131] reported PSCs with a PCE of 11.75% based on Ag:Al alloy electrode. Interestingly, this value is slightly higher than for the reference solar cells with Ag electrodes (11.45%) and much higher than for devices with Al electrodes (7.95%). In addition, Ag:Al alloy electrode showed a higher thermal stability as compared to Ag alone due to the formation of a passivation layer (AlO*_x_*) between the PCBM and the Ag:Al electrode, which prevented the diffusion of Ag as well as the penetration of moisture. The adhesion between Ag:Al and the active layer was also improved, thereby allowing the desired Ohmic contact at the electrode/PCBM interface through the formation of an ultrathin AlO*_x_* tunneling interlayer.

#### 2.3.3. Carbon Electrodes

Carbon materials have attracted an always increasing interest due to their highly tunable structures, low cost, abundant sources, and an attractive combination of properties, such as excellent electrical conductivity, chemical stability, and high carrier mobility [18]. Carbon electrode materials have been mainly divided into three categories, including conductive graphite/carbon black, graphene, and carbon nanotubes (CNTs), previously described in “Carbon-Based HTMs” Section 2.2.

It should be emphasized that organic HTMs and metal electrodes are the main causes for low PSC stability. Therefore, developing HTM-free PSCs with carbon electrodes (C-PSCs) is a promising approach to improve the device stability so as to allow commercialization of perovskite PV technology because carbon-based materials are cheap, stable, chemically resistant to halides, and inherently moisture tolerant [18,133].

Grancini et al. [134] fabricated 10 × 10 cm^2^ HTM-free perovskite solar modules by a fully printable industrial-scale process based on an ultra-stable 2D/3D (HOOC(CH_2_)_4_NH_3_)_2_PbI_4_/CH_3_NH_3_PbI_3_ perovskite junction and on a hydrophobic carbon black/graphite composite electrode. Interestingly, the module delivered a 11.2% efficiency stable for 410,000 h with zero loss in performances measured under controlled standard conditions and in the presence of oxygen and moisture.

Barichello et al. [135] reported a fully printable C-PSC using a homemade mesoporous insulating alumina (Al_2_O_3_) layer and a graphite/carbon black electrode. A water pre-treatment was applied prior to the perovskite deposition and growing and allowed enhancing the average open circuit voltage by +7% and the average PCE by +16% with respect to cells without water pre-treatment, with a final maximum PCE of 12.3%.

Finally, Tountas et al. [136] successfully developed small-area (0.04 cm^2^) and large-area (1 cm^2^) low-temperature processed mesoscopic HTL-free printable C-PSCs with a 2D perovskite electron-blocking layer between the perovskite and the carbon counter electrode. Reference devices were fabricated using a medium-temperature processed (MTP) carbon paste, while targeted cells were prepared using a low-temperature processed (LTP) graphene-based carbon paste. Interestingly, small-scale devices showed an increase in the PCE from 14.99% in the reference cell based on MTP counter electrodes to 17.68% for LTP ones, while large-area cells exhibited a PCE enhancement from 12.24 to 15.01%.

## 3. Fabrication Techniques

**Figure 6:**
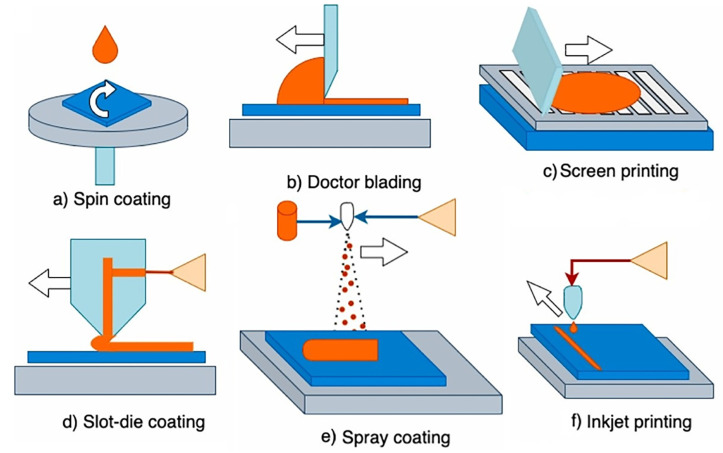
Main solution-based techniques for perovskite absorber layer deposition. Adapted with permission from Ref. [138] under the Creative Common license CC BY.

Solution- and vapor-based techniques are the main methodologies for producing perovskite absorber layers, the vapor-assisted method providing better film uniformity. Solution-processed deposition is performed using various techniques such as spin coating, doctor blading, screen printing, slot-die coating, spray coating and inkjet printing (see Figure 6). Vapor-based techniques, instead, are further classified into two main categories, namely physical- and chemical-based [137].

Vapor deposition process produces highly crystalline and uniform nanometer-thick films compared to micrometer-thick films using solution-processed techniques. Furthermore, vapor-deposited thin films are uniform, whereas solution-processed layered thin films exhibit much larger crystal grain sizes than the field of view. The main advantages of vapor deposition processes over solution processing techniques are that multilayer films can be produced over large areas and charge collection at interfaces can be easily tuned by using vapor deposition processes. Vapor deposition is therefore one of the preferred methods for producing solar cell layers of uniform thickness [139]. The shortcoming of vapor-based techniques is the requirement of vacuum. In vapor-based techniques, vacuum is used to increase the mean free path of the vapors for producing highly uniform thin films of very high purity [137]. The main solution- and vapor-based PSC fabrication techniques are thoroughly reviewed in the following paragraphs.

### 3.1. Solution-Based Techniques

#### 3.1.1. Spin Coating

Spin coating is the simplest and most cost-effective solution-processed technique for uniform deposition of perovskite layers of the PSCs. This technique is mainly exploited to produce small-area solar cells.

Spin coating is divided into one-step and two-step methods. In the one-step method, a mixture of PbX_2_ (X = Cl, I, Br) and AX (A = MA, FA, Cs) is dissolved in a polar aprotic solvent such as N, N-dimethylformamide (DMF) and dimethyl sulfoxide (DMSO) [140]. The perovskite film is formed by spin coating the synthesized perovskite precursor solution onto the substrate [141]. In the two-step method, films are deposited by separately synthesizing two different precursors; using this method, the control of the process is easier compared to the one-step method [142].

Typically, the films are baked after spin coating to produce a well-crystallized perovskite layer, as baking results in strong adhesion and bonding between metal cations and halogen anions [143]. By adjusting spin speed, acceleration, and spin coating time, film thickness and quality can be optimized.

In 1998, Liang et al. [144] first reported the preparation of PSCs using a two-step spin coating method, while the highest efficiency recorded at laboratory scale using this technique was about 22.1% [145].

The spin coating technique can be employed to fabricate inverted as well as regular PSC structures. The efficiency achieved with this technique is very high with a good level of reproducibility and morphology control in case of smaller sizes of modules, conversely this technique has the drawback of not producing a uniform film on larger areas [137]. Moreover, due to the slow processing speed and consistent material wastage, this technique is not a good solution for large-scale PSC production [146].

#### 3.1.2. Doctor Blading

Doctor blading, also known as blade coating, is a cost-effective, efficient, and simple method to produce films. In this technique, a continuous and uniform wet film is formed after scraping of the precursor solution dropped on the substrate [147]. Then, the film transforms into a perovskite film after annealing [148]. Doctor blading consistently reduces the consumption of precursor solution, requiring only 10 to 20 μL for a film with an area of 2.25 cm^2^, much less than the spin coating method (50–100 μL), thus clearly highlighting a higher utilization ratio of raw materials [147]. Therefore, it is suitable for large-scale industrial production due to its simplicity and cost savings. The thickness and quality of blade-coated films can be optimized by varying the distance between scraper and substrate, the speed and shape of scraper, the concentration of precursor solution, and the wettability of substrate, etc. [149].

Yang et al. [150] reported the first fully blade coated PSC in 2015. To improve the solubility and slow down the crystallization rate, the researchers added 1,8-diiodooctane (DIO) as an additive to the precursor solution, creating a dense and uniform perovskite film with large grains. In addition, the whole manufacturing process was carried out in ambient environment, so the impact of humidity variation on device performance was thoroughly investigated. As the humidity gradually decreased from 70%, the PCE markedly increased from 0.53 to 10.71%, which is very close to that exhibited by the device prepared by spin coating in glove box (11.32%). Moreover, flexible PSCs with efficiency of 7.14% were fabricated on PET substrate through the same method. The device also displayed a good stability, namely 80% of the initial PCE was maintained after bending for 100 times with a curvature radius of 1 cm. This work clearly demonstrated the feasibility of blade coating method to achieve high-performance PSCs.

In another work, Tang et al. [151] introduced compositional engineering in (MAPbI_3_)_0.6_(FAPbI_3_)_0.4_ precursor solution to reduce the substrate temperature during blade deposition. Interestingly, the addition of a small amount of Cs^+^ (less than 5 mol%) and Br^−^ (2.5 mol%) into the perovskite precursor solution was found to enhance phase purity at a lower blade temperature of 120 °C. In addition to composition engineering in the perovskite precursor solution, 5 mol% of excess methylammonium chloride (MACl) was added into the precursor solution to improve film coverage and eliminate pinholes, resulting from a delayed crystallization. The blade coated PSCs showed a PCE of 19.5% with a *J_sc_* of 23.1 mA/cm^2^, a *V_oc_* of 1.09 V, and an FF of 77%.

#### 3.1.3. Screen Printing

Screen printing is a widely employed film deposition technique in which a mesh (screen) is used to transfer a paste (ink) onto a flat substrate. The printed patterns are determined by the open mesh apertures of the screen. Unprinted areas are made impermeable to the paste by a blocking stencil. The paste is placed on the unprinted areas, and a printing squeegee is moved across the surface of the screen to fill the open mesh apertures with paste. The paste that is in the mesh apertures is pumped or pressed by capillary action to the substrate in a controlled and prescribed amount (the printed wet film is proportional to the thickness of the mesh and/or stencil). As the squeegee moves toward the back of the screen, the tension of the mesh pulls the mesh up away from the substrate (called snap-off), leaving the paste on the substrate surface [152].

For screen printing, the deposition area can reach several square meters, and the material utilization can be as high as 100% for a continuous process. The production capacity can be determined by the subsequent drying and/or sintering process, not limited by the screen printing process. The reproducibility can be affected by the characteristics of the paste and the screen voltage variations. In addition to the above-mentioned advantages, screen printing is also a low-cost and low-consumption technique that can provide PSCs with low production costs [152].

A triple mesoporous carbon-based hole transport layer (HTL)-free PSC (C-PSC) stack fabricated at large scale A4 size modules was demonstrated, using commercially available screen printable pastes and using only 1.6 mL of perovskite solution per module [153]. As expected, patterning the TiO_2_ blocking layer (BL) led to the improvement of both *V_oc_* and FF due to the reduced contact resistance of the carbon/FTO connection compared to carbon/BL/FTO. A further improvement simply came from storage in the dark at ambient conditions, while no deterioration was observed even after hundreds of hours at 70% RH. Interestingly, the best module with patterned BL still yielded a PCE as high as 6.6% (6.3% stabilized) two months after production.

#### 3.1.4. Slot-Die Coating

Slot-die coating is one of the most promising techniques to fabricate large-scale PSCs through roll-to-roll strategy [154]. This methodology works similarly to blade coating and the main difference is that the scraper is replaced with a coating head composed of two metal sheets, which feeds precursor solution from the narrow gap [155].

The use of slot-die printing in PSCs manufacturing can help preventing precursor contamination during the coating process. This is due to the isolation of the solution in the feeding system, which offers an advantage in terms of film quality and repeatability. Film uniformity and thickness can be finely tuned by varying the precursor solution concentration and the relative speed of coating head. Meanwhile, this technology shows a high solution utilization rate and exhibits a greater tolerance towards solution viscosity, concentration, and composition. Furthermore, as a typical non-contact method, slot-die coating can avoid the scratch between the coating head and substrate especially on rough surfaces [156].

Hwang et al. [157] first fabricated PSCs using a two-step slot-die coating method. They discovered that the drying process, which employed high-pressure nitrogen, played an important role in obtaining a uniform PbI_2_ film. Meanwhile, increasing the annealing temperature facilitated the growth of perovskite grains. More specifically, a perovskite film with small round grains and poor uniformity formed at room temperature, while compact perovskite films with large grain size of about 1 μm were obtained at 70 °C. As a result, a PCE of 11.96% was achieved by slot-die coating, confirming its feasibility in PSCs production.

The feasibility of manufacturing modules using slot-die coating is raising increased interest after optimization of processing parameters. Lee et al. [158] integrated wind blade on slot-die coating and a PCE of 8.3% was obtained on a module with an active surface area of 10 cm^2^ by adjusting the additives ratio (PbAc_2_ to PbCl_2_). Combined with the laser scribing, Di Giacomo et al. [159,160] fabricated a PSC module with an area of 12.5 × 13.5 cm^2^. The average PCE obtained over a small area (30 × 30 mm^2^) was 16.4%, which is comparable with the one manufactured by spin coating.

#### 3.1.5. Spray Coating

Spray coating has attracted widespread attention as an effective method to prepare large-area perovskite films [161]. The precursor solution is initially atomized into small droplets, which are then sprayed onto the substrate with the help of a carrying gas, resulting in the formation of a uniform, compact, and continuous wet film, and finally crystallized into a perovskite film upon solvent evaporation by heating. A spray equipment is usually assembled with three main parts: an atomizing nozzle, an injection pumping system and a heating plate [147]. Depending on the operating mode of the nozzle, spray equipment can be classified into pneumatic, ultrasonic, and electro spraying, of which the first two types are more commonly used [162,163]. The quality of the perovskite film fabricated by spray coating can be influenced by a series of processing parameters, which include the distance and relative speed between nozzle and substrate, crystallization temperature, flow rate, viscosity and composition of the precursor solution, etc. However, the possibility of obtaining low-cost, high-quality perovskite films and a highly reproducible manufacturing process make spray coating a very suitable strategy for preparing large-scale PSCs [164].

Spray coating was first applied by Barrows et al. [165] for PSC fabrication in 2014. A precursor solution consisting of PbCl_2_: MAI was sprayed onto a heated substrate (75 °C), then CH_3_NH_3_PbI_3−x_Cl_x_ was formed after annealing at a low temperature of 90 °C. In this study, it was proved that perovskite film quality can be effectively tuned by the substrate temperature, which plays a major role in the film nucleation and growth. In addition, the film thickness can be adjusted by controlling the nozzle height and relative speed. Finally, a PCE of 11.1% was achieved on a device area of 0.025 cm^2^. This study demonstrated the feasibility of using spray coating as an effective fabrication method for PSCs.

Heo et al. [166] prepared a MAPbI_3−x_Cl_x_ film by a two-step spray coating method. A low volatility solvent, γ-Butyrolactone (γ-GBL), was introduced into the perovskite precursor solution. The relationship between perovskite crystal size and the solvent inward (*F_in_*) and outward (*F_out_*) flux was thoroughly studied. The bottom of the perovskite film couldn’t dry completely when *F_in_* ≫ *F_out_* (DMF:GBL = 7:3), resulting in a rough perovskite film. While *F_in_* ≪ *F_out_* (DMF:GBL = 10:0) led to rapid crystallization, forming small-grained perovskite films. Only when *F_in_* was close to or slightly higher than *F_out_* (DMF:GBL = 8:2), precursor solution could re-infiltrate into crystallized perovskite layer and dissolve the smaller grains, thereby creating a uniform film with improved quality after the recrystallization. Consequently, a PCE of 18.3% was achieved on a 2.5 × 2.5 cm^2^ PSC. Additionally, a small module (10 × 10 cm^2^) with the structure FTO/TiO_2_/MAPbI_3−x_Cl_x_/PTAA/Au was fabricated and a PCE of 15.5% was achieved on an effective area of 40 cm^2^ after process optimization.

#### 3.1.6. Inkjet Printing

Inkjet printing is a process that is similar to using a printer. More in detail, a small nozzle drips a tiny amount of solution onto a substrate at a specific speed, and the uniform droplets coalesce into a wet film by carefully adjusting the spacing. Similarly to spray coating and slot-die coating, it is a non-contact, simple and low-cost film deposition method featured by a high utilization ratio of precursor solution. Moreover, specific patterns can be printed directly without the need for a mask by precisely tuning the size and position of droplets [147].

Inkjet printing can be divided into two categories based on the solution delivery method: continuous inkjet (CIJ) and on-demand inkjet (DOD) printing [167]. DOD is a reliable and cost-effective industrial technology widely used for printing electronic and optoelectronic materials, such as metal nanoparticles, polymers, PSCs, etc. [168]. The properties of the film can be adjusted by varying the nozzle distance to substrate, velocity, droplets size and patterns.

Wei et al. [169] exploited the scalable inkjet printing technology to fabricate PSCs with FTO/TiO_2_/MAPbI_3_/carbon configuration. A two-step deposition method was adopted, firstly spin coating the PbI_2_ film and subsequently inkjet printing the MAI/carbon ink. The resultant PSCs, based on the mixed MAI/carbon ink, exhibited a markedly reduced charge recombination due to enhanced interfacial contact between MAPbI_3_ and the carbon layer compared to the counterpart based on infiltrated MAI on FTO/TiO_2_/PbI_2_/carbon ink. The inkjet printed PSCs showed a PCE of 11.60% with a *J_sc_* of 17.20 mA/cm^2^, a *V_oc_* of 0.95 V, and an FF of 71%.

Li et al. [170] reported the effects of printing table temperature and MACl additive in precursor solution on crystal growth in correlation with photovoltaic performance. The inkjet printed PSCs showed a PCE of 12.3% with a *J_sc_* of 19.55 mA/cm^2^, a *V_oc_* of 0.91 V, and an FF of 69%.

### 3.2. Vapor-Based Techniques

#### 3.2.1. Physical Vapor Deposition (PVD)

PVD covers a wide range of film deposition techniques such as evaporation, laser ablation deposition, vacuum arc deposition and many different modes of physical sputter deposition [171]. PVD processes usually involve individual atoms or small clusters of atoms which are not normally found in the gas phase. Typically, these atoms are removed from a solid or liquid source, pass through an evacuated chamber, and impinge on a solid surface at which point the atoms stick and form a film. The ways to remove the atoms from the original source can be by heating the source or energetic particle bombardment by electrons, atoms, ions, molecules or photons. PVD differs from chemical vapor deposition (CVD) in that the main source of deposited species is solid or liquid, unlike a gas, and takes place at a vapor pressure much below the working pressure of the deposition system.

In 2013, Liu et al. [172] first reported the fabrication of perovskite films (MAPbI_3−x_Cl_x_) using PVD. More specifically, the perovskite thin films were deposited onto a spiro- OMeTAD substrate using the dual-source co-evaporation approach: the organic source used was MAI, while the inorganic source was PbCl_2_. Perovskite films produced by PVD were compared to those fabricated through spin coating method, and their X-ray diffraction (XRD) analysis confirmed that both the thin films showed an orthorhombic crystal structure. Additionally, the perovskite films manufactured by the vapor deposition method were more uniform and denser compared to the spin coated ones. Finally, PSCs fabricated through PVD method exhibited a PCE of 15.4%, thereby demonstrating the feasibility of using PVD for the production of PSCs.

Ono et al. [173] exploited PVD technique to fabricate large-area (5 × 5 cm^2^) semi-transparent perovskite films, reaching a PCE of 9.9%. It is worthwhile mentioning that the semi-transparent perovskite films with a thickness of approximately 50 nm can be easily fabricated through PVD, while it is challenging to achieve such thin perovskite layers through spin coating. The successful fabrication of large-area perovskite films represents a significant step forward in advancing the commercialization process of this technology.

#### 3.2.2. Chemical Vapor Deposition (CVD)

**Figure 7:**
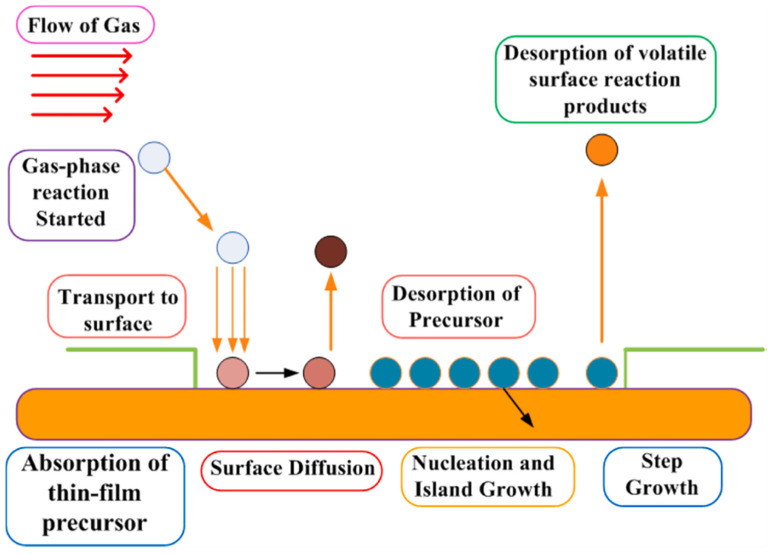
Basic steps involved in CVD technique. Adapted with permission from Ref. [174], Copyright 2023 Elsevier.

CVD is a process in which semiconducting materials and thin-films are prepared exploiting the chemical reaction of vapor-phase precursors. The manufacturing of perovskite thin films using CVD technique is schematically depicted in the diagram reported in Figure 7. Production involves evaporation and transport of reactants through gas flow into the reactor’s quartz tube. The reactions between intermediate ions occur in the reaction zone of the quartz tube. The precursor formed after the reaction is transported to the substrate and subsequently is adsorbed onto the substrate surface from thin film surface diffusion, nucleation and chemical reaction. The unreached fragments flow out of the reaction zone of the chamber to minimize the unreacted impurities [174].

In 2015, Fan and co-workers [175] reported an easy one-step CVD to deposit MAPbI_3_ and MAPbI_3–x_Cl_x_ perovskites. Inorganic (PbI_2_ or PbCl_2_) and organic sources (i.e., MAI) were loaded in the high-temperature zone in positions determined by their sublimation temperatures. Meanwhile, the substrates were placed in the low-temperature zone. During the deposition process, a carrier gas (i.e., Ar) was constantly flowed from the source toward the substrate to facilitate the chemical reaction. MAPbI_3_ and MAPbI_3–x_Cl_x_ films with large grains (>1 µm) and long carrier lifetime were deposited on substrates and, once employed in PSCs, allowed achieving a PCE in the range of 9–11%.

#### 3.2.3. Hybrid Vapor Deposition

Hybrid chemical vapor deposition process has been developed to fabricate PSCs based on methylammonium lead iodide (MAPbI_3_) and formamidinium lead iodide (FAPbI_3_) [176]. In this method, perovskite films are formed in two stages. In the first step, a lead halide film such as PbI_2_, PbCl_2_ or PbBr_2_ is deposited through thermal evaporation. In the second step, the as-prepared lead halide film is reacted with organic halide species under a controlled vapor atmosphere and pressure to form perovskite films inside a tube furnace.

Yokoyama et al. [177] proved the advantageous role of kinetically regulated gas-solid reactions in the fabrication of CH_3_NH_3_SnI_3_ thin films by the introduction of a low-temperature vapor assisted solution method. Compared to the one-step process, this method allowed fabricating compact films with excellent surface coverage. Furthermore, it has been demonstrated that by using this technique, the short-circuit behaviour frequently observed in conventional one-step manufacturing perovskite devices can be effectively avoided. However, there is a lack of in-depth research on this two-step vapor deposition method for inorganic CsPbBr_3_ PSCs.

### 3.3. Other Techniques

#### 3.3.1. Sol-Gel Method

The sol-gel method is a bottom-up synthesis technique commonly adopted to produce a wide range of materials, including inorganic membranes, monolithic glasses and ceramics, thin films, and ultra-fine powders. Today, it is even used to synthesize 1D nanomaterials [178].

The basis of the sol-gel method is to produce a homogeneous sol from the precursors and transform it into a gel. The solvent present in the gel is then removed from the gel structure and the remaining gel is dried. The properties of dried gel largely depend on the drying method. In other words, the “solvent removal method” is chosen based on the application for which the gel will be used [179].

The sol-gel method highlights many advantages over traditional processing technologies, including low reaction temperatures, precise composition control, high purity levels, and the ability to create processes for large-area applications [178].

Within this framework, in 2013 Grätzel and co-workers [180] applied for the first time a two-step sol-gel process to fabricate PSCs with a noticeable PCE above 15%. The two-step sol-gel process allowed an easy control of the surface morphology of perovskite films and the reduction of the defect content of the films. For the two-step sol-gel process, the perovskite is in direct contact with the residual PbI_2_ film and not with the porous TiO_2_ layer to avoid direct contact between the mesoporous TiO_2_ layer and the HTL layer. Although the direct contact between the perovskite and the residual PbI_2_ film increases the contact resistance, this is beneficial for PSCs when the perovskite light-absorber layer is not well fabricated.

#### 3.3.2. Electrodeposition

Electrodeposition is a versatile and roll-to-roll compatible technique used for the manufacturing of PSCs [137]. Its cost-effectiveness, rapidness, and high degree of uniformity make it a desirable technique for the deposition of perovskite layers [181].

Unlike spin coating, this technique does not require heating of the substrate because heating will produce a rough film. When heat is applied, film rupture and island formation occur randomly on the surface of the substrate [182]. The thin films produced by electrodeposition are very uniform with large-area coverage and the absence of sheer forces [183]. Deposition of perovskite layer on complex shaped substrates, which is not possible with the other techniques mentioned so far, makes it a very attractive approach for large-scale production [181].

#### 3.3.3. Laser Ablation

The laser ablation process is a subtractive method that allows fabricating micropatterns through the removal (ablation) of a small fraction of a substrate material under a focused pulsed laser beam.

A group of materials scientists from the University of Rome Tor Vergata, in collaboration with researchers in Germany, exploited the laser ablation technique to reduce the cell-to-module losses in perovskite solar modules [184]. The scientists investigated the layer structure of planar PSCs in three patterning steps, i.e., P1, P2 and P3, and determined the width of the perovskite cells to electrically isolate the two from each other by separating the two contact layers with P1 and P3. An absorber layer was obtained by maintaining the P2 structuring between P1 and P3 to provide an electrical interconnection.

To achieve a higher cell-to-module efficiency ratio, the area between P1 and P3 was kept as small as possible to avoid the challenges of preserving the integrity of the edge regions of the absorber layer during P2 patterning. By optimizing the laser design, establishing a relationship between geometrical fill factor, cell area width, and P1–P2–P3 laser parameters, a 20 × 20 cm^2^ minipanel was fabricated showing 11.9% stabilized efficiency and 2.3 W on an active area of 192 cm^2^, among the highest reported in literature at this size [184].

## 4. Discussion

### 4.1. Stability Enhancement Strategies

**Figure 8:**
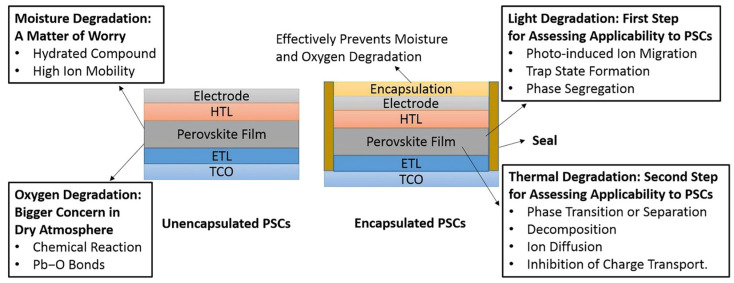
Mechanisms of degradation caused by external environmental factors for perovskite absorber. Reproduced with permission from Ref. [131], Copyright 2021 American Chemical Society.

One major obstacle to PSCs’ widespread use is represented by their reduced stability, mainly arising by the exposure of the perovskite absorber layer to moisture, oxygen, light, and heat (see Figure 8). Significant efforts have been devoted to stabilizing perovskite materials, including composition regulation, crystallization control, and interface optimization.

Perovskite materials play a significant role in improving PSC stability [131]. In this review paper, the latest approaches to enhance the perovskite layer stability in PSCs have been thoroughly examined. One key tactic to improve device stability is to envelop the susceptible perovskite absorber in layers of protection. Protective layers can be made of metal oxides (like TiO_2_, SnO_2_, and ZnO), polymers (like polyvinylpyrrolidone), and organic/inorganic hybrids (like SiO_2_-TiO_2_). By keeping oxygen and moisture out of the perovskite layer, these protective layers can prevent its deterioration. Making a perovskite absorber that is more resilient is another strategy. Additives that regulate perovskite crystallization and defect passivation can help to achieve this goal. For instance, Zhang and Zhu [185] have employed additives like guanidinium thiocyanate (GSCN) and 4-tert-butylpyridine (tBP) to increase the stability of PSCs. Besides these tactics, researchers have focused their efforts in creating new ways to enhance PSC stability. For instance, they improved long-wavelength light absorption with embedded dielectric nanoparticles by designing a new rear electrode and module structure for bifacial perovskite modules.

### 4.2. Materials Modification and Optimization

Optimizing and modifying materials is essential to raise PSCs’ stability and efficiency. The subsequent sections cover the latest research on charge transport layer engineering and doping, electrode interfacial modification and passivation, and perovskite composition and morphology control. Producing solar cells that are both highly efficient and stable requires careful control over the composition and morphology of the perovskite layer. The goal of morphology control is to generate large grain sizes, high immaculateness, enhanced crystallinity, and compact, pinhole-free films. This is usually achieved by using fast deposition-crystallization techniques, adding additives to the precursor solution, or modifying the annealing conditions to enhance perovskite crystal growth [185]. Additionally, scientists have focused on creating cutting-edge techniques to increase PSCs stability. In order to fabricate stable PSCs, doping and charge transport layer engineering are essential. Promising charge transport materials include metal oxides like NiO, CuO, Cu_2_O, MoO_3_, VO_x_, CuI, CuSCN, NiO, CuO, Cu_2_O, and NiCo_2_O_4_ [71,77,186]. It has been demonstrated that metal cation doping works well in PSCs to regulate charge transport and lower charge recombination. Enhancing the stability and performance of PSCs through electrode interfacial modification is a viable approach. Interface engineering can enhance device stability, interfacial defect passivation, carrier transport dynamics, and energy band alignment with regard to electrode interfacial modification materials. To enhance device performance, researchers have created a multifunctional interfacial material between tin oxide and perovskite.

### 4.3. Device Encapsulation and Protection

Device encapsulation and protection are critical for increasing the stability of PSCs. The following sections will discuss the most recent research on glass encapsulation and edge sealing and on polymer encapsulation and coating.

#### 4.3.1. Glass Encapsulation and Edge Sealing

PSCs can be made more stable through two promising techniques: glass encapsulation and edge sealing. Silicon solar cells with glass encapsulation have maintained 95% PCE after 20 years in use. A novel dual laser beam glass frit sealing method, ideal for hermetically encasing PSCs, was developed by Martins et al. [187]. More in detail, n-i-p PSCs employing PTAA as HTL were encapsulated using this technique and thermal cycling tests and conditions of elevated humidity were used to examine their long-term stability. After 500 h of humidity aging at 85% RH and 50 temperature cycles between −40 and 65 °C, no differences in performance were evidenced.

#### 4.3.2. Polymer Encapsulation and Coating

Promising techniques to increase the stability of PSCs also include polymer encapsulation and coating. The main strategy for enhancing stability consists in modifying a component of the PSC, such as the HTL or even the perovskite absorber layer itself. CF_4_ plasma treatments, perovskite grain crosslinking, moisture-resistant HTLs, and different polymeric encapsulation techniques have all been used to increase moisture stability. The use of fluorinated encapsulation techniques has proven especially effective, producing cells that maintained their PCE after 75 days at 50% RH and 5 mW cm^−2^ of ultraviolet (UV) radiation [130,188].

### 4.4. Environmental and Operational Factors

Sustaining stability over the long term is one of the hardest challenges for PSCs to accomplish because the perovskite layer is easily degraded by operational and environmental variables like temperature, humidity, and light intensity. Therefore, it is essential for PSCs to continue to be developed and commercialized to comprehend how these factors affect PSC performance and durability as well as to develop effective mitigation strategies.

#### 4.4.1. Humidity and Moisture Effects and Mitigation

It is commonly known that humidity and moisture are the most harmful elements to the stability of PSCs because they can lead to the dissolution and disintegration of the perovskite layer, the corrosion and delamination of the electrodes and charge transport layers, and the development of flaws and contaminants in the device. A number of factors, such as the composition of the perovskites, the design of the device, the surrounding environment, and the length of exposure, affect the mechanisms and effects of humidity and moisture on PSCs. The composition of the perovskite, the addition of hydrophobic additives or dopants, the application of protective coatings or encapsulants, and the optimization of device engineering and fabrication are some of the strategies that have been suggested to increase the moisture stability of PSCs [189]. Nevertheless, these approaches often entail balancing PCE, scalability, and moisture stability of PSCs. Therefore, more investigation is needed to create fresh, practical methods for enhancing PSCs’ moisture stability without sacrificing their usefulness or performance.

#### 4.4.2. Temperature and Thermal Effects and Mitigation

Other significant variables that impact the stability of PSCs include temperature, the expansion and contraction of the device’s component parts, thermal stress, and device fatigue. Numerous factors, such as the composition of the perovskite, the design of the device, the circumstances of thermal cycling, and the length of exposure, influence temperature and the thermal effects and mechanisms on PSCs [190].

#### 4.4.3. Light Illumination and Photodegradation Effects and Mitigation

In addition, photoinduced degradation and decomposition of the perovskite layer, photooxidation and photoreduction of the electrodes and charge transport layers, and the creation of defects and impurities in the device are significant factors impacting PSC stability. The composition of the perovskite, the design of the device, the light’s intensity and spectrum, and the length of the exposure period are some of the factors that influence the effects and mechanisms of light illumination and photodegradation on PSCs. Because they have the potential to create a multitude of internal degradation pathways and mechanisms, environmental and operational factors are also crucial for the stability of PSCs [191]. In this review, the latest developments and difficulties in PSCs in terms of three main environmental and operational factors, i.e., light illumination and photodegradation, temperature and thermal fluctuations, and humidity and moisture, have been summed up. In order to better illustrate the review’s primary ideas, a few figures and tables have been incorporated. With regard to the fabrication and use of PSCs, we sincerely hope that this review can provide some insightful viewpoints.

## 5. Conclusions and Outlook

This review paper focuses on the structural and fundamental aspects of PSCs that have resulted in considerable performance gains. High electron mobility, a long diffusion wavelength, and a high absorption coefficient are examples of important electrical and optical qualities. PSC synthesis methods are discussed, with solution-based production being the most cost-effective and widely used industrial method. Over the last decade, researchers have made great progress toward increasing the efficiency and stability of PSCs. Despite these developments, the stability of PSCs remains a significant hurdle to their widespread implementation, as PSCs are particularly sensitive to moisture due to the fundamentally weak connections in the perovskite structure. Manufacturing scalability is also a challenge, as the spin coating/drop casting techniques employed in most laboratory-scale testing are unsuitable for large-scale manufacturing.

To solve these difficulties, researchers are studying new materials and production techniques. Some of the future developments being researched are more ecologically friendly lead-free PSCs and high-efficiency multi-junction cells. More research is needed to improve the intrinsic chemical stability of halide perovskites. Researchers should focus on creating creative strategies to improve PSC stability under extreme conditions such as high temperatures and long-term illumination. Collaboration among academics from diverse disciplines, such as materials science, physics, chemistry, and engineering, can help to speed advancement in this subject.

It can be concluded that PSCs have made remarkable progress in the past decade, but still face several obstacles that need to be overcome before they can be widely deployed and commercialized. Some of the major problem statements and possible pathways for future research are:

- Developing novel and environmentally friendly perovskite materials and compositions that can achieve high efficiency, stability, and reproducibility, while avoiding the use of toxic or scarce elements.
- Optimizing the device architecture and interface engineering to enhance the charge transport, extraction, and collection, as well as to reduce the parasitic losses and degradation mechanisms.
- Scaling up the fabrication techniques from lab to fab, using industry-compatible and scalable methods that can produce large-area and uniform PSCs with high yield and quality.
- Improving the stability of PSCs under various operating and environmental conditions, such as temperature, humidity, light, oxygen, and mechanical stress, by employing effective encapsulation and protection methods, as well as designing self-healing and adaptive PSCs.
- Evaluating the environmental impact and life cycle assessment of PSCs, as well as addressing the lead toxicity and recycling issues, by following the principles of green chemistry and circular economy.

Finally, the list of problem statements and potential solutions has been made apparent. This will assist the scientific community in focusing this review paper on the most important problems in the field and identifying the areas that require more investigation. By doing this, the authors will be able to further the development of this exciting technology and significantly improve the field of PSCs.

## Figures and Tables

**Table 1:** Examples of perovskite structures with different symmetries. The crystal structure of BaTiO_3_ has been reproduced with permission from Ref. [17], Copyright 2018 Elsevier, while the crystal structure of CH_3_NH_3_PbI_3_ has been adapted with permission from Ref. [19], Copyright 2018 Elsevier.

Compound	Structure	Symmetry
CaTiO_3_	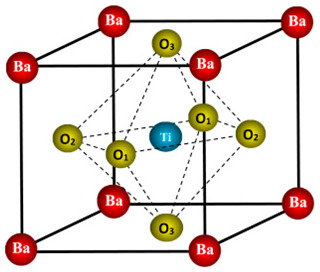 Replace Ba with Ca or Sr	Orthorhombic, cubic
BaTiO_3_		Cubic, tetragonal, orthorhombic, rhombohedral
SrTiO_3_		Cubic
CH_3_NH_3_PbI_3_	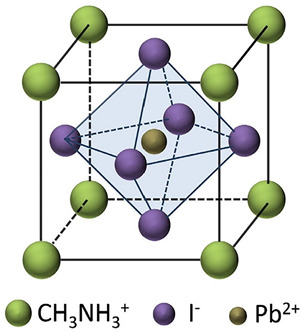	Tetragonal, cubic

**Table 2:** Examples of the electronic properties of perovskites and their solar cell efficiencies.

Compound	Band Gap (eV)	Carrier Mobility (cm^2^ V^−1^s^−1^)	Defect Density (cm^−3^)	Solar Cell Efficiency (%)
BaTiO_3_	2.7–3.2	0.01–0.1	10^18^–10^20^	-
SrTiO_3_	3.2	0.1–10	10^16^–10^18^	-
CH_3_NH_3_PbI_3_	1.5–1.6	10–100	10^15^–10^16^	25.2

**Table 3:** Examples of composition engineering of perovskites with different A, B, and X ions. The crystal structure of CsPbI_3_ has been reproduced with permission from Ref. [26], Copyright 2016 Elsevier; the crystal structures of MAPbI_3_ and FAPbI_3_ have been adapted from Ref. [27] under the Creative Common license CC BY; the crystal structure of MASnI_3_ has been adapted with permission from Ref. [29], Copyright 2021 Wiley; the crystal structure of MAPbBr_3_ has been adapted with permission from Ref. [30], Copyright 2020 American Chemical Society; the crystal structure of MAPbCl_3_ has been adapted with permission from Ref. [31], Copyright 2022 American Chemical Society.

Compound	Structure	Band Gap (eV)
CsPbI_3_	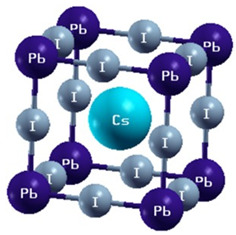	1.73
MAPbI_3_	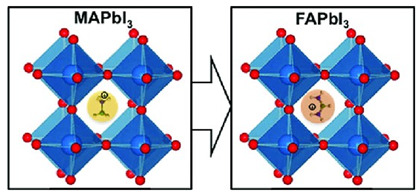	1.55
FAPbI_3_		1.48
MASnI_3_	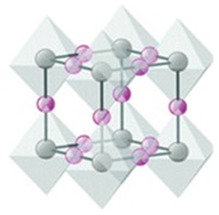	1.30
MAPbBr_3_	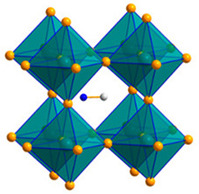	2.30
MAPbCl_3_	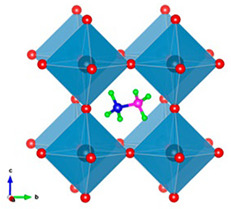	3.10

## Data Availability

Not applicable.
